# Salbutamol modifies the neuromuscular junction in a mouse model of ColQ myasthenic syndrome

**DOI:** 10.1093/hmg/ddz059

**Published:** 2019-04-01

**Authors:** Grace M McMacken, Sally Spendiff, Roger G Whittaker, Emily O’Connor, Rachel M Howarth, Veronika Boczonadi, Rita Horvath, Clarke R Slater, Hanns Lochmüller

**Affiliations:** 1 John Walton Muscular Dystrophy Research Centre, Institute of Genetic Medicine, Newcastle University, Newcastle Upon Tyne, UK; 2 Children's Hospital of Eastern Ontario Research Institute, University of Ottawa, Ottawa, Canada; 3 Institute of Neuroscience, Newcastle University, Newcastle Upon Tyne, UK; 4 Department of Clinical Neurosciences, University of Cambridge, Cambridge, UK; 5 Department of Neuropediatrics and Muscle Disorders, Medical Center – University of Freiburg, Faculty of Medicine, Freiburg, Germany; 6 Centro Nacional de Análisis Genómico (CNAG-CRG), Center for Genomic Regulation, Barcelona Institute of Science and Technology (BIST), Barcelona, Catalonia, Spain; 7 Division of Neurology, Department of Medicine, The Ottawa Hospital, Ottawa, Canada

## Abstract

The β-adrenergic agonists salbutamol and ephedrine have proven to be effective as therapies for human disorders of the neuromuscular junction, in particular many subsets of congenital myasthenic syndromes. However, the mechanisms underlying this clinical benefit are unknown and improved understanding of the effect of adrenergic signalling on the neuromuscular junction is essential to facilitate the development of more targeted therapies. Here, we investigated the effect of salbutamol treatment on the neuromuscular junction in the ColQ deficient mouse, a model of end-plate acetylcholinesterase deficiency. ColQ^−/−^ mice received 7 weeks of daily salbutamol injection, and the effect on muscle strength and neuromuscular junction morphology was analysed. We show that salbutamol leads to a gradual improvement in muscle strength in ColQ^−/−^ mice. In addition, the neuromuscular junctions of salbutamol treated mice showed significant improvements in several postsynaptic morphological defects, including increased synaptic area, acetylcholine receptor area and density, and extent of postjunctional folds. These changes occurred without alterations in skeletal muscle fibre size or type. These findings suggest that β-adrenergic agonists lead to functional benefit in the ColQ^−/−^ mouse and to long-term structural changes at the neuromuscular junction. These effects are primarily at the postsynaptic membrane and may lead to enhanced neuromuscular transmission.

## Introduction

Motor neurons contact their target muscle fibres at highly specialised chemical synapses, neuromuscular junctions (NMJ). The NMJ is the pathogenic target in a wide range of human diseases, including those resulting from genetic defects affecting a diverse range of proteins which are critical for synaptic function, the Congenital Myasthenic Syndromes (CMS) (1,2). CMS arise from mutations affecting presynaptic, synaptic or postsynaptic proteins at the NMJ, resulting in impairment of neuromuscular transmission by one or more mechanisms. A precise molecular classification of CMS subtype is of great importance for the diagnosis and genetic counselling of patients, but also to allow administration of effective treatment as different drugs may be beneficial or deleterious depending on the CMS subtype (3). For many subtypes, clinical benefit is gained from acetylcholinesterase (AChE) inhibitors, which augment the synaptic response to acetylcholine (ACh) (4). However, AChE inhibitors are ineffective or even detrimental in Dok7 CMS, slow-channel CMS, end-plate AChE deficiency and MuSK CMS.

Ephedrine, a sympathomimetic with α- and β-adrenergic effects, and salbutamol, a selective β_2−_agonist, have been successfully used to treat many patients with CMS subtypes which are not effectively treated by anticholinesterases. These include those with mutations that cause deficits in Dok-7, Agrin, MuSK, ALG2, AChR ( $\varepsilon$ -subunit) and in end-plate AChE deficiency (5–10). In contrast with the effects of anticholinesterases, the full effects of these adrenergic treatments are not immediate, reaching a peak only after several months (5,11). Among the varied pharmacologic effects of β-agonists, there is considerable evidence for their numerous effects in regulating skeletal muscle structure and function, and in exerting an anabolic effect on skeletal muscle protein metabolism (12–16). These actions are predominantly mediated through the β_2_ receptors (ADBR2), and involve cAMP signalling (17). While these effects were initially exploited by the livestock industry, their use quickly expanded to include body builders and athletes. In more recent years, experimental interest has further expanded to trial the treatment of a wide range of muscle-wasting and neuromuscular diseases. In animal and human studies, β_2_-adrenergic agonists have been reported to have a positive but limited effect in dystrophic and injured muscle, as well as in congenital myopathies and fascioscapulohumeral muscular dystrophy (18–21). In addition, further studies have suggested β_2_-agonists may have a modest effect in spinal muscular atrophy, and in denervated muscle following spinal cord injury (22,23).

It is in treatment of CMS however, that sympathomimetics have demonstrated conclusive clinical benefit, and they now comprise standard treatment for some subtypes of CMS in the form of oral ephedrine or salbutamol (24). Several observational studies demonstrate improvements in motor symptoms and timed tests when CMS patients are administered ephedrine or salbutamol and mobility may often improve to the extent of regaining ambulation in wheelchair bound patients (7,9–11,25–27). However, it is not known why treatments acting via pathways mediated by the sympathetic nervous system have therapeutic benefit in disorders of the NMJ. Despite its potential implications in the understanding of both the pathogenesis and treatment of many neuromuscular diseases, the effect of β-agonists on the maturation and maintenance of NMJs has never been clearly defined.

In order to address this, we have studied the effect of salbutamol treatment on a model of end plate AChE deficiency, the ColQ knockout (ColQ^−/−^) mouse. In these mice, endplate AChE deficiency is caused by mutations not in *ACHE* itself but in *COLQ*, which encodes the collagenic tail subunit bound to the catalytic subunit in the asymmetric AChE found at the skeletal NMJ (28,29). The N-terminal domain of ColQ anchors asymmetric AChE to the synaptic basal lamina (30). In humans, mutations in *COLQ* typically result in fatigable muscle weakness presenting in the neonatal period or early infancy, often accompanied by episodes of respiratory failure (31). The NMJs of these patients have abnormalities in both the function (prolonged response to ACh due to the absence of AChE activity) and structure (disrupted postsynaptic apparatus, probably resulting from excessive Ca^2+^entry into the muscle through the AChRs) (32,33).

Generally, these patients show either no long-term benefit or worsening of symptoms with AChE inhibitors. However, treatment with salbutamol and ephedrine has been shown to lead to improved mobility and respiratory function in these patients (25). ColQ^−/−^ mice also lack all asymmetric AChE and exhibit both muscle weakness and abnormalities of the NMJ (34,35), thus closely resembling the human disease. In addition to its role in anchoring AChE, ColQ binds to the key postsynaptic organiser protein MuSK, and ColQ has also been shown to have an important regulatory role in postsynaptic development and maturation through this interaction (36–38). Given the clinical improvement from salbutamol treatment in CMS patients is gradual, requiring a period of weeks to months for full benefit, we have tested the effects of long-term administration of salbutamol, i.e. administered over a period of 7 weeks, in the ColQ^−/−^ mouse.

Here, we show that salbutamol treatment leads to a gradual improvement in muscle strength in the ColQ^−/−^ mouse. In addition, we show that morphological defects of the NMJ in the ColQ^−/−^ mouse can be partially rescued by salbutamol treatment, in ways which are likely to lead to enhanced neuromuscular transmission. These results provide new evidence for the long-term effects of adrenergic signalling on the structural properties of the NMJ.

## Results

### Salbutamol treatment improves muscle strength in ColQ^−/−^ mice

**Figure 1:**
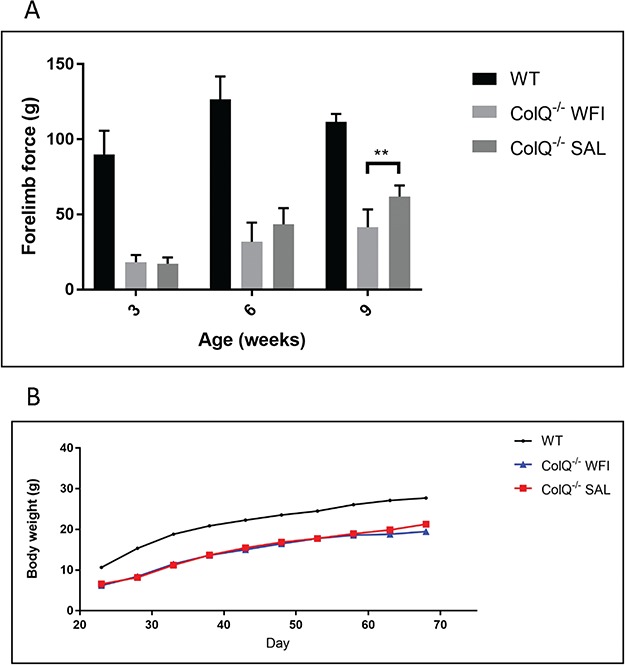
Salbutamol treatment improves muscle strength in ColQ^−/−^ mice but does not alter body weight. **A.** Forelimb grip strength of WT, water treated ColQ^−/−^ (ColQ^−/−^ WFI) and salbutamol treated ColQ^−/−^ (ColQ^−/−^ SAL) mice measured at 3, 6 and 9 weeks of age. Grip strength is significantly impaired in water treated ColQ^−/−^ mice compared to WT littermates at baseline, and at subsequent time points. Salbutamol treated ColQ^−/−^ mice showed a tendency towards improved grip strength after 3 weeks of treatment (6-wks-old) which became statistically significant after 6 weeks of treatment (9-wks-old). n = 6 animals per group. Error bars depict S.D. ^**^ indicates *P* < 0.01. **B.** Growth curve demonstrating changes in body weight over 7 weeks of salbutamol treatment. ColQ^−/−^ mice are significantly smaller than WT littermates at all time-points and body weight is not affected by salbutamol treatment. n = 6 animals per group.

From 3 weeks of age, ColQ^−/−^ mice received daily subcutaneous injections of salbutamol or vehicle control (water) for 7 weeks. Previous work has shown that ColQ^−/−^ mice exhibit muscle weakness which is apparent from P5 (34). We used forelimb grip strength to assess muscle strength at 3 time points (Fig. 1A) in wild type (WT), ColQ^−/−^ mice treated with water and ColQ^−/−^ mice treated with salbutamol. The performance of water treated ColQ^−/−^ mice was significantly worse than WT littermates at baseline (3 weeks old) and at subsequent measurements (6 weeks old and 9 weeks old). In comparison, ColQ^−/−^ mice treated with salbutamol showed a gradual improvement in grip strength, which became significantly better than water treated ColQ^−/−^ mice at 9 weeks (after 6 weeks of salbutamol treatment) (water treated ColQ^−/−^ 41.6 ± 11.7 g vs. salbutamol treated ColQ^−/−^ 61.9 ± 7.4 g (mean ± S.D.)). ColQ^−/−^ mice were also smaller than WT littermates, having a body weight approximately 50% that of littermates at 3 weeks (Fig. 1B) (34). This low body weight persisted into adulthood, and was not altered by salbutamol, with no significant difference in salbutamol treated ColQ^−/−^ mice body weight during 7 weeks of salbutamol treatment (water treated ColQ^−/−^ 21.3 ± 2.71 g vs. salbutamol treated ColQ^−/−^ 19.5 ± 2.13 g). In summary, salbutamol led to a gradual improvement in limb muscle strength but did not affect body weight in ColQ^−/−^ mice.

### Salbutamol treatment improves NMJ structural defects in ColQ^−/−^ mice

**Figure 2:**
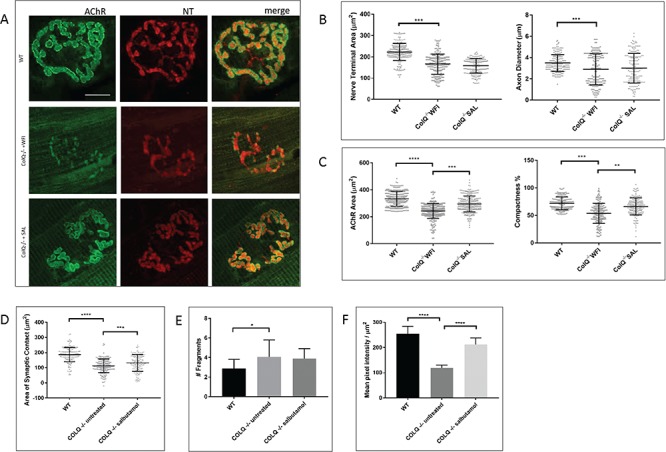
Salbutamol treatment improves postsynaptic NMJ morphology in ColQ^−/−^ mice. **A.** Representative confocal micrographs of NMJs in the lumbrical muscles of 10-wk-old WT, water treated ColQ^−/−^ (ColQ^−/−^ WFI) and salbutamol treated ColQ^−/−^ (ColQ^−/−^ SAL) mice, labelled with anti-neurofilament (red) and anti-synaptophysin (red, both NT) and Alexa fluor 488 α-bungarotoxin (green, AChR). Water treated ColQ^−/−^ mice display variable NMJ morphology with smaller NMJs with reduced AChR density. Scale bar, 10 μm. **B.** Quantitative analysis of presynaptic variables from lumbrical muscles. ColQ^−/−^ mice exhibited smaller nerve terminals with thinner axons, and these variables were unchanged in salbutamol treated mutants. **C.** Quantitative analysis of postsynaptic variables from lumbrical muscles. Water treated ColQ^−/−^ mice had reduced AChR area and compactness, which were significantly increased in salbutamol treated ColQ^−/−^ mice compared to water treated ColQ^−/−^ mice. **D.** Quantitative analysis of area of synaptic contact. ColQ^−/−^ mice have significantly reduced area of synaptic contact which is increased by salbutamol treatment but remains smaller than in WT animals. **E.** Quantitative analysis of fragmentation of NMJs. ColQ^−/−^ NMJs exhibit significantly increased number of AChR rich fragments per NMJ, and this was not different in salbutamol treated animals. n = 180 NMJs analysed per group for B-E. Analysis of NMJ-morph variables from soleus muscle are provided in Supplementary Material, Figure S2. **F**. Quantification of α-bungarotoxin fluorescence intensity of individual NMJs measured from projection of confocal stacks from lumbrical muscles. Fluorescence intensity was significantly decreased in water treated ColQ^−/−^ mice, and was significantly increased in salbutamol treated ColQ^−/−^ mice compared to water treated ColQ^−/−^ mice. n = 120 NMJs analysed per group for F. All error bars depict S.D. Unpaired Student’s *t* tests. ^∗∗∗∗^p < 0.0001, ^∗∗∗^p < 0.001, ^∗∗^p < 0.01, ^∗^p < 0.05.

It has previously been shown that the ColQ^−/−^ mouse exhibits abnormal synaptic structure, including NMJs which appear fragmented or immature (34). We studied the pre and postsynaptic morphology of NMJs from 10 week old ColQ^−/−^ lumbrical muscles and compared these to salbutamol treated ColQ^−/−^ muscles, using a standardised morphometric analysis platform (‘NMJ-morph’) (39). ColQ^−/−^ mice did demonstrate presynaptic structural defects, with small but significant decreases in axon diameter (WT 3.49 ± 0.79 μm vs water treated ColQ^−/−^ 2.90 ± 1.47 μm) and nerve terminal area (WT 227.16 ± 35.12 μm^2^ vs water treated ColQ^−/−^ 166.28 ± 47.16 μm^2^) (Fig. 2B). These presynaptic defects were not significantly different in salbutamol treated ColQ^−/−^ mice (axon diameter and nerve terminal area 2.96 ± 1.43 μm and 159.42 ± 33.89 μm^2^ respectively).

Postsynaptic morphology was more obviously perturbed in the ColQ^−/−^ mice (Fig. 2C-E and Supplementary Material, Fig. S1), with ColQ^−/−^ lumbrical muscles showing significantly reduced AChR area (WT 332.117 ± 58.55 μm^2^ vs. water treated ColQ^−/−^ 236.91 ± 63.13 μm^2^) and reduced NMJ ‘compactness’ (derived from (AChR Area/Endplate area) x100), a measure of AChR dispersal (WT 71.77 ± 13.49% vs. water treated ColQ^−/−^ 54.30 ± 19.01%) (39,40). These postsynaptic defects were significantly improved in salbutamol treated mice (AChR area 303.52 ± 75.69 μm^2^ and compactness 76.23 ± 22.26%).

Furthermore, the area of synaptic contact (the area of contact between pre and postsynaptic NMJ components) was significantly reduced in the ColQ^−/−^ mice compared to WT littermates (Fig. 2D) (WT 191.61 ± 46.69 μm^2^ vs. water treated ColQ^−/−^ 93.8 ± 64.98 μm^2^), and this was increased by salbutamol treatment (141.63 ± 58.65 μm^2^). In keeping with previous studies, we found the number of discrete fragments of AChR rich membrane was significantly increased in the ColQ^−/−^ mice (WT 2.88 ± 0.93 fragments vs. water treated ColQ^−/−^ 4.10 ± 1.74 fragments). Fragmentation was not altered by salbutamol treatment, however (3.91 ± 1.00 fragments). We also examined these parameters in soleus muscles (Supplementary Material, Fig. S1). Salbutamol treatment also improved AChR area, compactness and area of synaptic contact in soleus muscles, and we found no difference in the magnitude of the effect of salbutamol in soleus muscle compared to lumbricals (Supplementary Material, Fig. S1).

These results prompted us to examine whether the fluorescence intensity per μm^2^ of α-BTX, a measure of the local AChR density, was different in salbutamol treated mice. In lumbricals from water treated ColQ^−/−^ mice, AChR density was significantly reduced compared to WT littermates (mean pixel intensity per μm^2^ WT 255.39 ± 149.49 vs. water treated ColQ^−/−^ 119.68 ± 69.49). In comparison, in salbutamol treated ColQ^−/−^ lumbricals, AChR density was significantly increased compared to water treated ColQ^−/−^ mice (mean pixel intensity per μm^2^ 211.89 ± 139.41).

Together, these observations suggest that salbutamol treatment can alter NMJ structural defects in ColQ^−/−^ mice, and in particular increase AChR area, synaptic area and AChR density.

### Salbutamol alters ultrastructural appearances of the postsynaptic membrane

**Figure 3:**
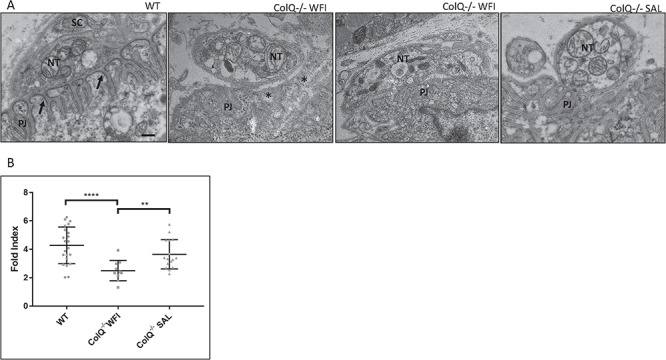
The extent of postjunctional folding is increased in salbutamol treated ColQ^−/−^ mice. A. Representative electron micrographs of sections through single boutons from intercostal muscles of 10-wk-old WT, water treated ColQ^−/−^ (ColQ^−/−^ WFI) and salbutamol treated ColQ^−/−^ (ColQ^−/−^ SAL) mice. NT: nerve terminal, PJ: postjunctional folds, SC; Schwann cell. In WT mice, the postsynaptic membrane is extensively infolded and AChRs can be seen as areas of high electron-density at the crests of the folds (arrows). In ColQ^−/−^ mice, the appearance of increased membrane density at the tops of the folds is lost, the extent of folding is reduced and the synaptic space appears widened (stars, right panel) although some NMJs appear normal (left panel). Salbutamol treated ColQ^−/−^ NMJs exhibit similar ultrastructural appearances to WT littermates. Scale bar 0.5 μm. B. Quantitative analysis of changes in the extent of folding with salbutamol treatment. In salbutamol treated ColQ^−/−^ mice, the extent of postjunctional folding is significantly increased compared to water treated ColQ^−/−^ mice, as measured by fold index (fold length/postsynaptic length). n ≥ 15 boutons from three mice analysed per group. Mann Whitney *U* test. ^∗∗∗∗^p < 0.0001, ^∗∗^p < 0.01.

In mature NMJs, the postsynaptic membrane is invaginated with extensive folds extending into the postsynaptic membrane, which amplify the transmitter action of ACh (41). AChRs accumulate at the crests of these postjunctional folds, which can be visualised at the EM level by electron dense material at the top of the folds and extending partly down the folds (42). Under the electron microscope, many NMJs from intercostal muscles of 10 week old water treated ColQ^−/−^ mice appeared normal, as has been previously reported in 6 month old ColQ^−/−^ mice (34). However, in 50% of NMJs the extent of folding appeared clearly reduced and regions of high electron-density at the crests of folds were lost (Fig. 3A). We quantified the extent of postjunctional folding using fold index (total surface length of postsynaptic membrane measured along the tops of the folds/total length of postsynaptic membrane including folds) a measure of the increase in postsynaptic membrane area resulting from folding (43). This revealed a significant reduction in extent of folding in ColQ^−/−^ mice compared to WT (mean fold index WT 4.28 ± 1.38 vs. water treated ColQ^−/−^ 2.50 ± 0.72) (Fig. 3B). In addition, in salbutamol treated ColQ^−/−^ mice, fold index was significantly increased compared to water treated ColQ^−/−^ mice (3.65 ± 1.03). Additional features measured from EM images are provided in Supplementary Material, Table S1.

### Immunoreactivity of Agrin, Dystroglycan and MuSK in ColQ^−/−^ mice

The effects of salbutamol on postsynaptic structural defects prompted us to explore the effect of salbutamol on MuSK, agrin and α-dystroglycan. MuSK provides the primary scaffold for AChR clustering and postsynaptic differentiation (44). The C-terminus of ColQ binds MuSK and ColQ deficiency leads to reduced levels of membrane bound MuSK (36,37). The glycoprotein agrin is secreted by motor axon terminals to activate the LRP4/MuSK/Dok7 complex to induce and stabilise AChR clusters (45). Dystroglycan, a component of the dystrophin associated glycoprotein complex (DGC), is essential for the assembly of a synaptic basement membrane, and linking the extracellular matrix to the cytoskeleton (46). ColQ binds to perlecan which in turn binds α-dystroglycan, and this interaction is necessary for the synaptic localisation of AChE (47). In addition, the localisation of AChRs at the crests of the folds arises through their interaction with the DGC (48).

**Figure 4:**
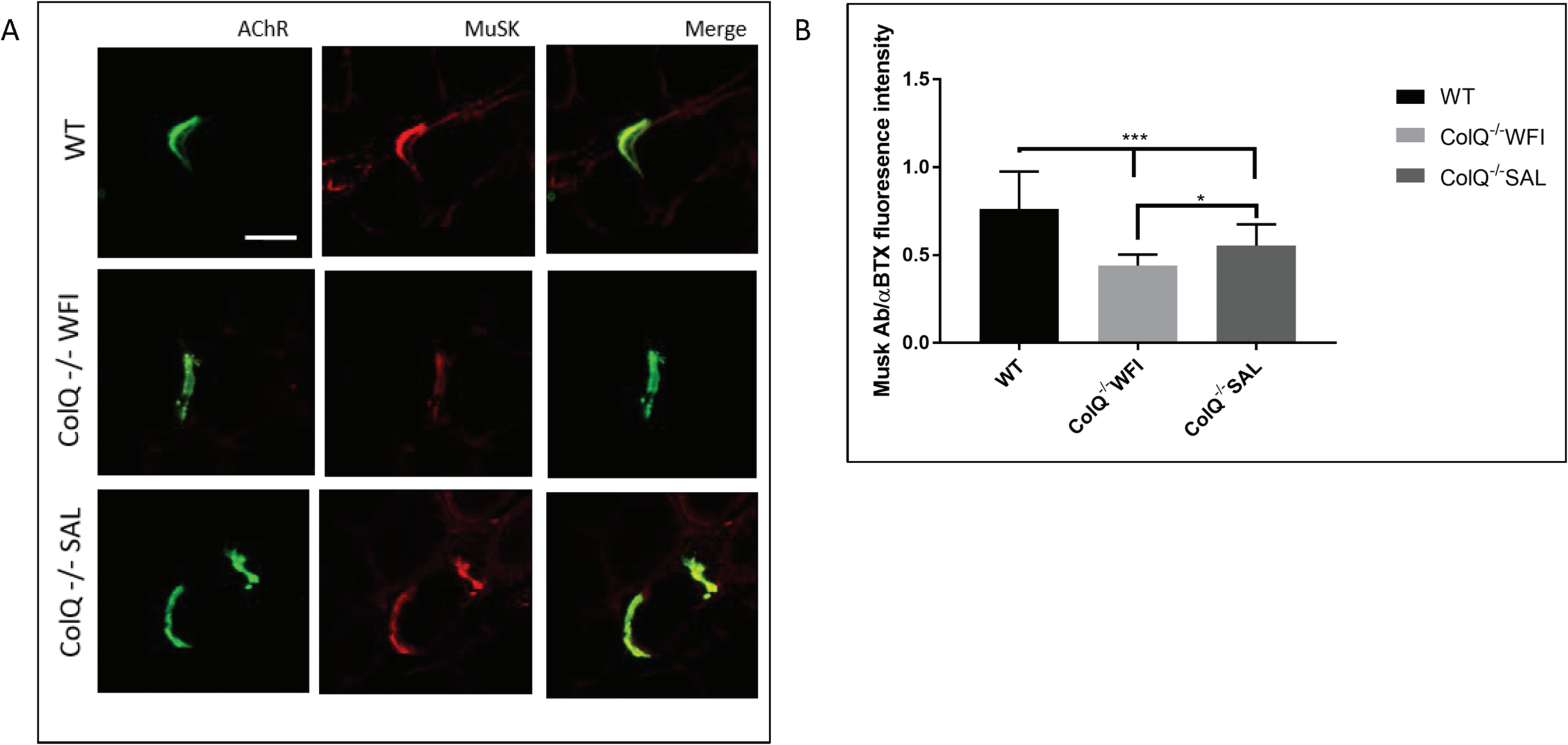
Salbutamol treatment alters MuSK immunoreactivity in ColQ^−/−^ mice. **A.** AChR and MuSK clusters labelled with Alexa 488 α-BTX (green, AChR) and anti-MuSK antibody (red) respectively on transverse sections of gastrocnemius muscle from 10-wk-old WT, water treated ColQ^−/−^ (ColQ^−/−^ WFI) and salbutamol treated ColQ^−/−^ (ColQ^−/−^ SAL) mice. Scale bar, 20 μm. **B.** Quantification of MuSK fluorescence intensities from projection of confocal stacks. MuSK fluorescence intensity per AChR cluster was decreased in ColQ^−/−^ muscle, and significantly increased in salbutamol treated ColQ^−/−^ mice compared to water treated. n = 180 NMJs per group for B. Unpaired Student’s *t* tests. ^∗∗∗^p < 0.001, ^∗^p < 0.05, n.s. not significant.

In order to determine membrane bound MuSK protein levels, we analysed MuSK fluorescence intensities per AChR cluster in transverse sections of gastrocnemius muscle from WT, water treated ColQ^−/−^ and salbutamol treated ColQ^−/−^ mice (Fig. 4A). MuSK fluorescence intensity was significantly reduced in water treated ColQ^−/−^ mice compared to WT (mean MuSK-to-α-BTX fluorescence intensity ratio WT 0.76 ± 0.21 vs. water treated ColQ^−/−^ 0.44 ± 0.06). In salbutamol treated ColQ^−/−^ muscle, the MuSK-to-α-BTX fluorescence intensity ratio was increased compared to water treated ColQ^−/−^ mice (Fig. 4B, mean MuSK-to-α-BTX fluorescence intensity ratio 0.55 ± 0.12 in salbutamol treated ColQ^−/−^ mice). In contrast to MuSK staining, the fluorescence intensity of agrin was not significantly different in either water treated ColQ^−/−^ or salbutamol treated ColQ^−/−^ mice compared to WT animals (data not shown). In addition, the distribution and intensity of immunostaining of α-dystroglycan was qualitatively similar in ColQ^−/−^ and WT mice, and was not affected by salbutamol treatment (data not shown).

### Salbutamol treatment does not alter muscle fibre area or fibre type composition in ColQ^−/−^ mice

**Figure 5:**
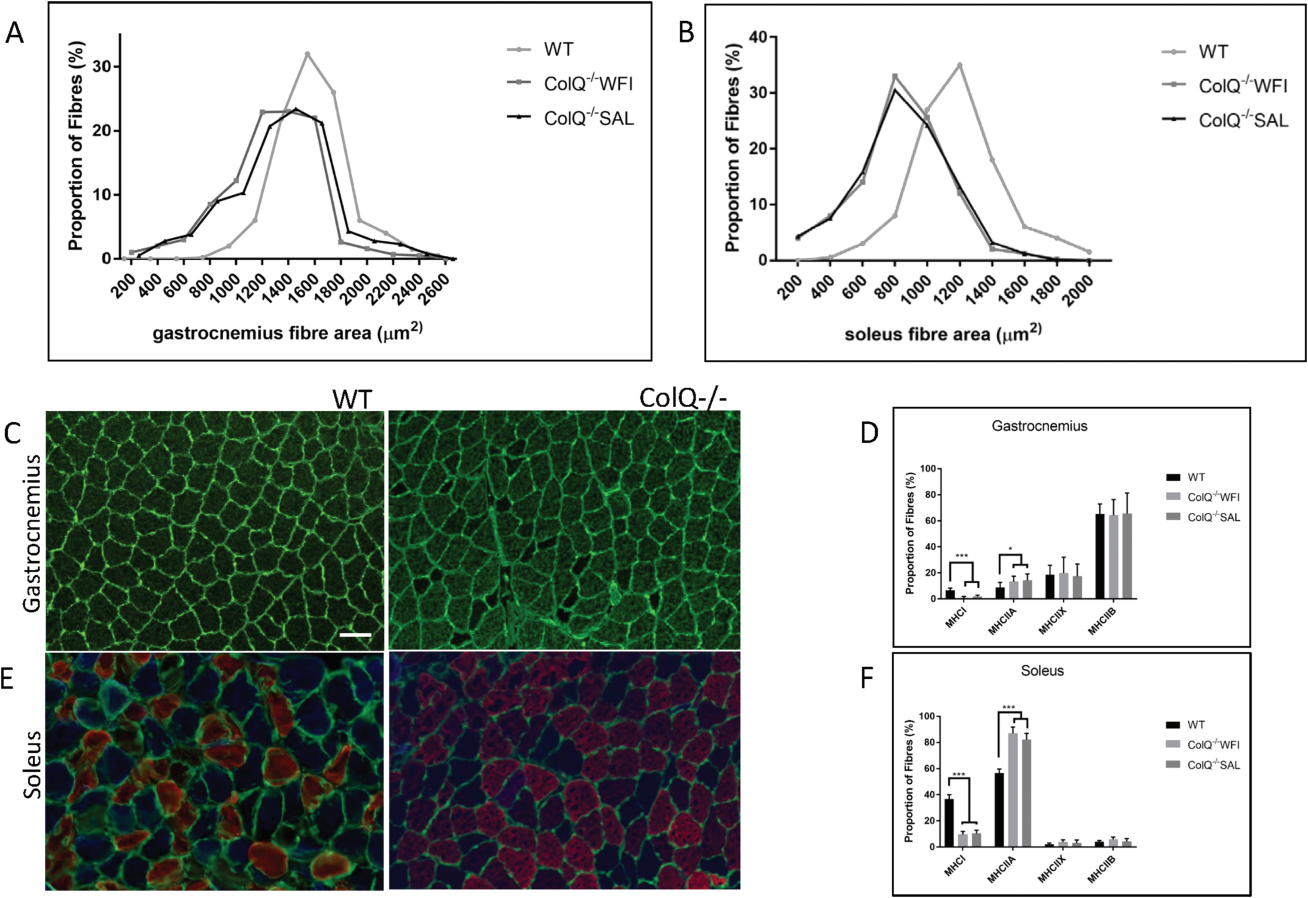
Alterations in muscle fibre diameter and type in ColQ^−/−^ mice are not affected by salbutamol treatment. **A, B:** Frequency distributions of muscle fibre cross sectional area from 10-wk-old WT, water treated ColQ^−/−^ (ColQ^−/−^ WFI) and salbutamol treated ColQ^−/−^ (ColQ^−/−^ SAL) gastrocnemius (A) and soleus (B) muscles. Plots represent percentage of fibres in each size bin. Fibres are significantly smaller in ColQ^−/−^ muscles compared with those in WT fibres (Mann-Whitney *U* test) but fibre diameter was unaffected by 7 weeks of salbutamol treatment. n = 6 muscles per genotype, with ≥500 muscle fibres sampled to generate the size distributions. ^∗∗∗^p < 0.001, ^∗^p < 0.05 **C—F:** Fibre type composition in WT and ColQ^−/−^ muscles. Transverse sections of gastrocnemius (C) and soleus (E) muscles from 10-wk-old WT and ColQ^−/−^ animals, stained with anti-laminin (green), to delineate the fibre circumference and MHC Type 1 (blue), Type IIa (red) and Type IIb (green). ColQ^−/−^ mice exhibit significantly fewer Type 1 fibres and increased Type 2A fibres in both gastrocnemius (D) and soleus (F) muscles, which was not affected by 7 weeks of salbutamol treatment. Scale bar, 50 μm.

It has previously been shown that ColQ^−/−^ mice exhibit both reduced muscle fibre diameter and alteration of fibre type composition (35,38). We therefore examined the effect of salbutamol treatment on these parameters. At 10 weeks, muscle fibre cross sectional area in water treated ColQ^−/−^ mice was significantly reduced in both gastrocnemius (mean cross sectional area WT 1740 ± 434.73 μm^2^ vs. water treated ColQ^−/−^ 1342 ± 317.51 μm^2^) and soleus (WT 1188 ± 105.4 μm^2^ vs water treated ColQ^−/−^ 781 ± 185.44 μm^2^). Muscle fibre area was not significantly different in salbutamol treated ColQ^−/−^ mice compared to water treated ColQ^−/−^ mice (Fig. 5A and B) (mean cross sectional area 1308 ± 350.45 μm^2^ and 802 ± 217.51 μm^2^ in gastrocnemius and soleus muscles respectively). In addition, examination of fibre type composition in gastrocnemius and soleus muscles revealed a dramatic reduction in MHC type 1 expressing fibres in both muscles, as well as an increase in MHC type 2A fibres in water treated ColQ^−/−^ mice. Again, these changes in fibre type composition were unaffected by salbutamol treatment (Fig. 5C-F). These data indicate that the effect of salbutamol on NMJ structure and on grip strength in the ColQ^−/−^ mouse is not secondary to changes in skeletal muscle trophism.

### Immunoreactivity of β2 adrenoceptors colocalises with the NMJ in WT and ColQ^−/−^ mice

**Figure 6:**
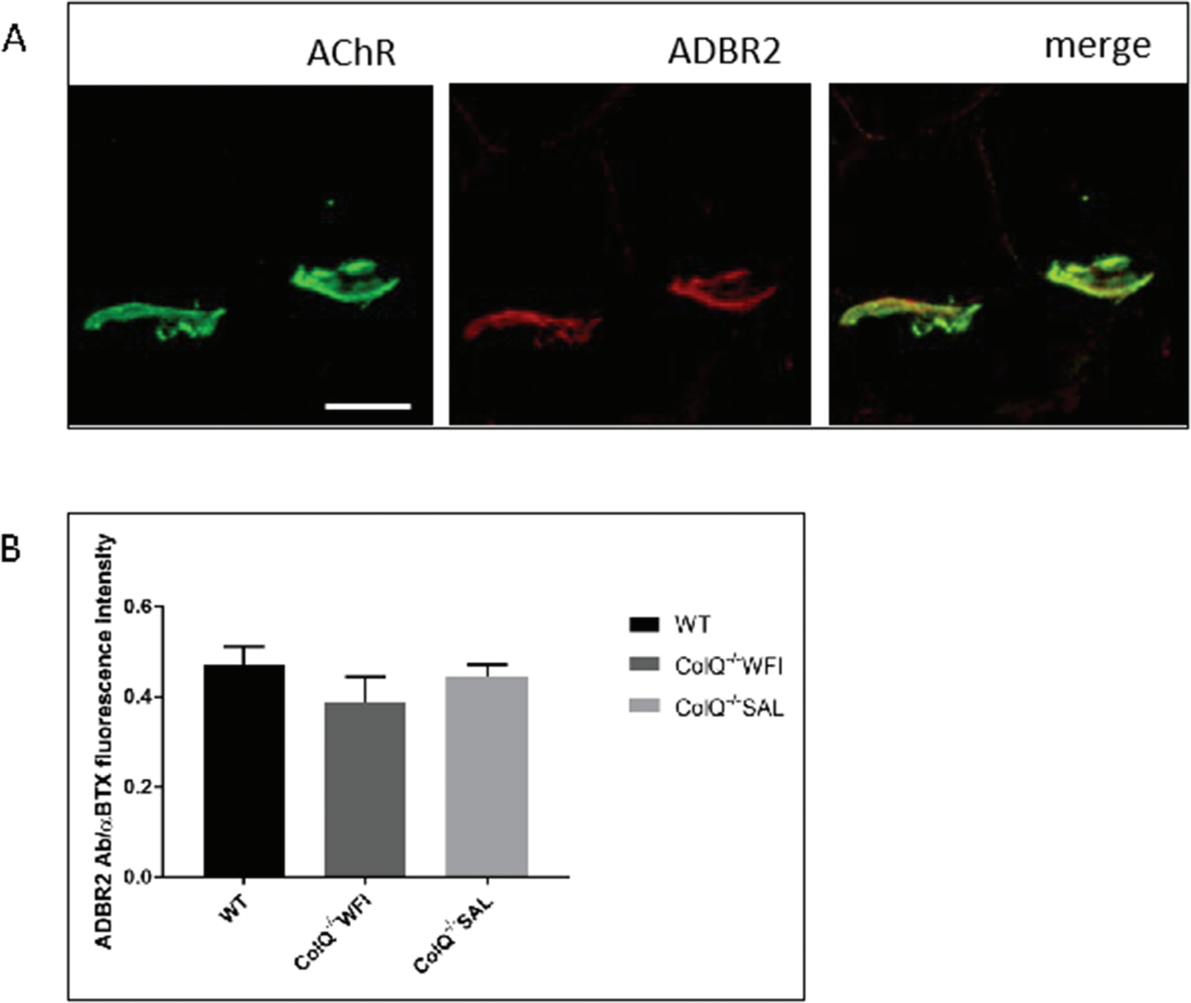
β_2_ adrenergic receptor immunostaining co-localises with the NMJ. **A.** Transverse sections of gastrocnemius muscle from 10-wk-old WT mice stained with anti-ADBR2 antibody (red) and Alexa 488 α-BTX (green, AChR). ADBR2 staining is enriched at the NMJ. Scale bar, 20 μm. **B.** Quantification of ADBR2 fluorescence intensities per AChR cluster from projection of confocal stacks. ADBR2 fluorescence intensity per AChR cluster was not significantly different between WT, water treated ColQ^−/−^ or salbutamol treated ColQ^−/−^ mice. Unpaired Student’s *t* tests.

Salbutamol is a selective ADBR2 agonist, and it has been previously shown that ADBR2 is the predominant adrenoceptor subtype in skeletal muscle (49,50). In order to investigate the distribution of ADBR2 on the muscle membrane we stained transverse sections of gastrocnemius muscle with ADBR2 antibody and α-BTX (Fig. 6A). This revealed co-localisation of ADBR2 immunoreactivity at the NMJ. In addition, this pattern of immunostaining did not appear to be altered in ColQ^−/−^ mice and was not affected by 7 weeks of salbutamol treatment (Fig. 6B).

## Discussion

Our study provides evidence that treatment of AChE-deficient ColQ^−/−^ mice with the sympathomimetic salbutamol, over a period of weeks, partially normalises both their weakness of grip strength and the structural abnormalities of NMJs that may help to explain that weakness. It has long been suspected that activation of adrenergic receptors can alter neuromuscular transmission. *In vitro*, ephedrine increases quantal content, and ephedrine and salbutamol block the AChR channel, although only when applied at higher concentrations than obtained at therapeutic doses in humans (51,52). In addition, adrenaline and noradrenaline can potentiate neuromuscular transmission, and activation of α_1_ and β adrenoceptors has been shown to enhance nerve evoked ACh release (53–55). However, these immediate effects do not account for the delayed therapeutic action of salbutamol in patients with CMS, which suggest an additional longer term modulation of NMJ function. There is, however, increasing evidence for the role of the sympathetic nervous system in maintenance of the NMJ. Recent studies demonstrate that NMJs are intimately linked to a network of sympathetic neurons within skeletal muscle which increase during postnatal development, a network which is critical for the morphological integrity of the NMJ (56,57). We previously showed that salbutamol rescued aberrant NMJ development in zebrafish embryos lacking Dok7 and MuSK (58). Here, in a setting more closely resembling human disease and treatment regimens, we show that long-term administration of salbutamol leads to functional benefit and improves postsynaptic structural defects in a mouse model of end plate AChE deficiency.

Salbutamol treatment resulted in a significant increase in grip strength of ColQ^−/−^ mice. This effect was gradual, becoming significantly better only after 6 weeks of daily administration. This mirrors the effect in humans with ColQ CMS where, unlike the rapid clinical benefit seen from AChE inhibitors in other forms of CMS, the response to sympathomimetics is more gradual with an increasingly positive response over 3–6 months of treatment (11,59). As previously reported, the phenotype of the ColQ^−/−^ mouse is not as severe as would be expected from a complete lack of AChE. Interestingly, in both the salbutamol treated and water treated ColQ^−/−^ mice, grip strength was improved at 9 weeks compared to 3 weeks. In previous studies of the ColQ^−/−^ mice, NMJ abnormalities also appeared to lessen with age, suggesting a compensatory mechanism (34). These studies suggested that capping of the motor nerve terminal by the Schwann cell serves to protect the end-plate from lost ACh clearance (34,60). However, invasion of the synaptic cleft by the Schwann cell was not a feature in any of the NMJs examined with EM in this study. The findings may reflect the homeostatic plasticity of the NMJ, as occurs in response to trauma, toxins and in autoimmune myasthenia gravis (41,61,62). The precise processes underlying adaptive plasticity in the ColQ^−/−^ mouse have not yet been determined.

The effects of β-adrenergic agonists on skeletal muscles are known to include muscle fibre hypertrophy and alteration of fibre type composition (63–65). The ColQ^−/−^ mouse exhibits features of muscle atrophy, with reduction in type I and increase in type IIA fibres as well as reduced fibre size (38). It is plausible therefore, that adrenergic agonists lead to clinical benefit in ColQ CMS due to promotion of muscle growth. However, our analysis of fibre type composition and area in both soleus and gastrocnemius muscles revealed no detectable effect of salbutamol treatment on any parameter measured in ColQ^−/−^ mice. This suggests that the improved grip strength in salbutamol treated ColQ^−/−^ animals cannot be readily explained by an anabolic effect on skeletal muscle. This is consistent with the lack of evidence of clinical benefit of sympathomimetic treatment in myopathies and muscular dystrophies (20,21,66,67). Since both the structure and function of the NMJs is abnormal in ColQ^−/−^ mice (34), we turned our attention to the possibility that salbutamol may act to enhance muscle activation by action on the NMJs.

In support of this view, we found evidence of a partial normalisation of synaptic area, local AChR density and the extent of postsynaptic folding in salbutamol treated ColQ^−/−^ mice, all changes which would be likely to enhance the efficacy of neuromuscular transmission. Using standardised analysis of NMJs (39), we confirmed pronounced defects in the structure of the NMJs in water treated ColQ^−/−^ mice which are likely to be associated with a decreased efficacy of neuromuscular transmission (34,35). These involved changes to both presynaptic and postsynaptic components of the NMJ. On the presynaptic side, there was a modest reduction in both the terminal axon diameter and nerve terminal area. There was also a significant reduction in the area of overlap between the nerve terminal and the underlying region of high AChR density (‘synaptic area’). There is an approximately linear relationship between synaptic area and quantal content, and a pathological reduction in synaptic area has been demonstrated in patients with ColQ CMS, and in CMS due to mutations in the postsynaptic adaptor *DOK7* (60,68,69). Therefore, any reduction in synaptic area is likely to be associated with reduced quantal content and the efficacy of neuromuscular transmission.

On the postsynaptic side, we found that at 10 weeks of age, the labelling intensity of AChRs was strikingly reduced in water treated ColQ^−/−^ mice, suggesting a reduced local AChR density at the NMJs. Any reduction in local AChR density would be expected to reduce the number of AChRs opened by individual transmitter quanta and thus impair neuromuscular transmission. However, previous studies of ColQ^−/−^ mouse showed an increased AChR density in sternomastoid and soleus muscles at 7 days of age (36). These divergent findings may be due to differences in the age of the mice examined, given that at postnatal day 7 mouse NMJs are still undergoing structural and molecular maturation and AChR rich membrane is not yet fully restricted to regions of motor axon contact (70). AChR deficiency has also been shown in ColQ CMS patients (60). In parallel with the reduction of AChR density, we found a decrease in the area occupied by a high density of AChRs. This extends previous studies showing reduced AChR area in adult (diaphragm, levator auris longis) and 7 days old (soleus and sternomastoid) ColQ^−/−^ mice (35,36).

A likely explanation for reduced AChR density at the NMJ is the simplification and disorder of the postsynaptic folds associated with AChE deficiency (32). Since a substantial fraction of AChRs are normally present in the membrane of the folds closest to the nerve, any disruption of membrane folding may result in reduced numbers of AChRs as detected by fluorescence microscopy. Consistent with this view, our preliminary analysis of NMJs from intercostal muscles of ColQ^−/−^ mice confirmed the significant reduction in the extent and orderliness of postjunctional folds. The high density of voltage-gated sodium channels in the depths of the folds is believed to facilitate initiation of the muscle fibre action potential (69,71), suggesting a second way in which the reduced folding in ColQ^−/−^ mice may impair neuromuscular transmission.

Treatment of ColQ^−/−^ mice with salbutamol for 7 weeks resulted in a partial normalisation of a number of the abnormalities of NMJ structure which are likely to be associated with impaired NMJ function, particularly those associated with the postsynaptic component. These include the area of synaptic contact, the local AChR density and the extent of folding, with similar degrees of effect found in lumbrical and soleus muscles. Whilst electrophysiological studies will be required in order to confirm that these structural alterations are coupled with improved NMJ function, the changes are likely to have important implications for the efficiency of neuromuscular transmission (41,72–74). Our findings of pronounced postsynaptic structural effects from salbutamol are in keeping with the fact that it is the CMS subtypes with predominant postsynaptic alterations which show greatest clinical benefit from salbutamol and ephedrine (4,7,11,75).

The differentiation of the postsynaptic region at the NMJ is strongly influenced by the activity of MuSK (44). ColQ deficiency has been shown to regulate membrane bound MuSK, and to subsequently lead to decreased signalling of MuSK as measured by reduced phosphorylation of the β-AChR subunit (36). This led us to ask whether salbutamol treatment might enhance the expression of membrane bound MuSK in ColQ−/− mice. We observed reduced MuSK immunoreactivity in gastrocnemius muscle from untreated ColQ^−/−^ mice, and this was significantly increased by salbutamol treatment. It is not possible to say whether salbutamol alters the activity of the MuSK signalling pathway, or whether this finding is secondary to improved postsynaptic architecture in salbutamol treated muscles. Further studies will be required to determine whether salbutamol affects MuSK phosphorylation and kinase activity.

The distribution of ADBR2 on skeletal muscle membrane has not been previously well described. Here we observed ADBR2 immunostaining precisely co-localising with AChRs in gastrocnemius muscle from both WT and mutant adult mice, similar to the patterns previously observed in one study of mouse extensor digitorum longus muscle (56). This co-localisation is intriguing and further suggests that ADBR2 and their signalling components have an important contribution to NMJ function.

In some cases of CMS, the clinical benefit from salbutamol seems to attenuate after years of treatment (unpublished observations). This may be due to desensitisation of ADBR2 after chronic agonist administration, as occurs when β_2_-adrenergic agonists are used in the treatment of chronic heart failure and asthma, although this has not been confirmed in CMS (76,77). The development of more targeted therapies for CMS, potentially those which act downstream of ADBR2, are therefore essential to improve quality of care. We previously showed that the effects of salbutamol at NMJs of zebrafish embryos could be blocked by a selective ADBR2 antagonist, and could be replicated by directly increasing cyclic AMP with forskolin (58). Identification of the cellular processes which are regulated by the cAMP and the cAMP-dependent protein kinase (PKA) signalling pathway at the NMJ is required for the identification of possible therapeutic targets.

Postsynaptic differentiation is controlled by factors released from motor nerves, glial cells and by intrinsic muscle signalling. There are thus a multitude of pathways through which activation of ADBR2 receptors and the cAMP signalling pathway could lead to improved postsynaptic NMJ morphology (78,79). Nonetheless, our observations in the ColQ^−/−^ mouse provide further evidence for important interplay between the NMJ and adrenergic signalling pathways. An understanding of the effect of sympathomimetics at the NMJ will be instrumental in order to facilitate the development of more targeted therapies, which benefit NMJ function whilst minimising systemic side-effects.

## Materials and Methods

### Mice

All procedures were approved by the Home Office and were carried out in accordance to the Animals Scientific Procedures Act of 1986 under project licence 70/8538. The generation of the ColQ^−/−^ mouse was described by Feng et al (34). ColQ^+/−^ mice were donated by the Krejci laboratory, COGNAC G cognition action group, Université Paris Descartes. Mice were bred and housed in the animal facility at the Functional Genomics Unit, Institute of Genetic Medicine, Newcastle University.

### Drug treatment of mice

From post-natal day 21, ColQ^−/−^ mice received daily subcutaneous injections for 7 weeks of either Salbutamol (α-[(tert-Butylamino)methyl]-4-hydroxy-m-xylene-α,α′-diol, Albuterol; Sigma) diluted in water for injection (n = 6 animals) or the equivalent volume of water alone (n = 6 animals). Injections were delivered into the loose skin over the interscapular area. Salbutamol was administered at a dose of 5 mg/kg which is equivalent to doses used in the treatment of CMS patients (59). Mice were weighed prior to injection daily. Wild type (WT) littermates were weighed and inspected daily in a similar manner to injected littermates. Researchers handling the animals were blinded to the genotype and drug allocation of each animal. Animals were weighed and inspected daily for signs of drug toxicity or side effects, and no adverse effects were observed. All mice were sacrificed within six hours of the last injection at 10 weeks of age.

### Forelimb grip strength test

An electronic grip strength meter (Bioseb) was used to determine the maximal peak force of the forelimbs (80). Mice were allowed to grasp the grid and were pulled horizontally by the tail until the grip was released. The pull force was measured when the pulling force overcame the mouse’s forelimb grip strength. Testing was performed in six animals per group at three time points—3 weeks, 6 weeks and 9 weeks of age. Three measurements were performed per mouse during each test and the average of these three measurements was used for statistical evaluation. These experiments were conducted in a blinded fashion by the same experimenter, 5 hours after injection.

### Whole-mount muscle staining

Whole soleus and hindlimb lumbrical muscles from 10 week old mice were dissected, washed in phosphate buffered saline (PBS) for 30 minutes, and then fixed (1% paraformaldehyde in 0.1 M PBS) for 10 minutes before being teased into small bundles. After teasing, muscles were fixed in 1% PFA overnight at 4 °C. Muscles were permeabilised in ethanol followed by methanol (10 min at −20 °C each), followed by an incubation/permeabilisation step in 5% horse serum, 5% BSA, 2% Triton X-100 (for 4 hours at room temperature). Muscles were incubated overnight with antibodies against neurofilament (mouse monoclonal anti-neurofilament, 1:200, Cell Signalling technology), and synaptophysin (rabbit polyclonal anti-synaptophysin, 1:100, Thermo Fisher Scientific) or against agrin (mouse polyclonal anti-agrin, 1:250, Abcam) in PBS containing 5% horse serum, 5% BSA. The next day muscles were washed in PBS containing 5% horse serum, 5% BSA for four hours and then incubated in Alexa Fluor® 488 α-bungarotoxin (α-BTX) (1:250, Thermo Fisher Scientific), Alexa Fluor® 568 goat anti-mouse (1:250, Thermo Fisher Scientific) and Alexa Fluor® 594 goat anti-rabbit (1:250, Life Technologies) overnight. The following day muscles were washed in PBS for 4 hours and then mounted on slides with Vectashield mounting medium for fluorescence microscopy (Vector Laboratories).

### NMJ imaging and analysis

Samples were visualised using a Nikon A1R laser scanning confocal microscope. Laser power and parameter settings were kept constant and Z-stack images (1-μm intervals) were acquired with x63 oil immersion objective and processed using NIS-elements AR 4.20.02 software. Soleus and lumbrical muscles from 6 mice per group were imaged; ≥30 NMJs were analysed per muscle. Variables for NMJ structural analysis were measured from maximum intensity Z-stack images using the ‘NMJ-morph’ protocol on ImageJ as described previously (39). For quantitation of AChR density, confocal micrographs of control and experimental mice were collected in the same session permitting comparison of fluorescence intensity. AChR density was performed only on lumbrical muscles on which NMJs could be visualised relatively quickly. The perimeters of clusters were delimited and the area and average pixel intensity calculated using ImageJ software. The AChR cluster outline was then placed in an adjacent area without clusters to record background fluorescence intensity. This reading was subtracted from the cluster reading, to give a background-corrected intensity. Lumbrical muscles from six mice per group were imaged; ≥20 NMJs were analysed per muscle.

### Immunostaining on sections

Immunostaining of ADBR2, MuSK and α-dystroglycan was performed on 10 μm transverse sections of gastrocnemius muscle cut using a cryostat (Microm HM 560, Zeiss) in the region of the motor end-plate. Sections were fixed in acetone at 4 °C for 15 minutes and then permeabilised in 0.1% Triton X-100 for 15 minutes at room temperature. Sections were blocked in PBS containing 10% goat serum, 1% BSA for 30 minutes and then incubated with primary antibody (rabbit anti-MuSK 1:500, Abcam; rabbit anti-ADRB2 1:200; Santa Cruz Biotechnology; mouse anti-α-dystroglycan 1:100; Santa Cruz Biotechnology) in blocking buffer for 2 hours at room temperature. Sections were washed and incubated in Alexa Fluor® 488 α-BTX (1:250), and secondary antibodies Alexa Fluor® 594 goat anti-rabbit (1:250) for 1 hour at room temperature. Sections were then washed and mounted using Vectashield mounting medium.

### Quantification of MuSK, agrin and ADBBR2 staining intensity

Quantification of relative staining intensities was performed as described previously (36,81). Transverse sections of gastrocnemius (MuSK and ADBR2 staining) or whole mounts of lumbrical muscles (agrin staining) were visualised using a Nikon A1R laser scanning confocal microscope. Laser power and parameter settings were kept constant and Z-stack images (1-μm intervals) were acquired with x63 oil immersion objective and processed using NIS-elements AR 4.20.02 software. Confocal micrographs of control and experimental mice were collected in the same session. Quantification of relative area and fluorescence intensity of α-BGT to MuSK, agrin or ADBR2 was performed using ImageJ software. The perimeters of clusters were delimited on the α-BGT channel and the area and fluorescence intensity measured. This selection was restored in the Alexa Fluor® 594 channel and the area and fluorescence intensity of MuSK, agrin or ADBR2 was measured. The background intensity in each channel was subtracted from the pixel intensity of the protein of interest, and the background-corrected intensity for each channel were divided to give a MuSK/agrin/ADBR2-to-α-BGT fluorescence intensity ratio. For MuSK and ADBR2 fluorescence intensity, gastrocnemius muscles from six mice per group were imaged; ≥40 NMJs were analysed per muscle. For agrin fluorescence intensity, lumbrical muscles from six mice per group were imaged and 20 NMJs were analysed per muscle. All imaged AChR clusters were measured.

### Fibre type identification

Transverse 10 μm sections of soleus and gastrocnemius muscles were cut using a cryostat and labelled for Myosin Heavy Chains (MHC) MHCI, MHCIIa, and MHCIIb and MHCIIx. Sections were blocked (10% normal goat serum in PBS) for 1 hour at room temperature and then incubated with primary antibodies: rabbit polyclonal IgG anti-laminin (Sigma 1:750), MHCI (BA-F8 Mouse monoclonal IgG2b) 1:25, MHCIIa (Sc71, Mouse monoclonal IgG1), MHCIIb (BF-F3 Mouse monoclonal IgM) 1:200 and MHCIIx (6H1 Mouse monoclonal IgM) 1:25, all DSHB, for 1 hour at room temperature. Sections were then washed in PBS and incubated in secondary antibodies for 1 hour, washed and mounted using Vectashield mounting medium. Images were captured using a Zeiss Axio Imager fluorescent microscope with Zen software and analysed using ImageJ software. In each section, every adjacent field was examined moving from left to right in a systematic manner until the required number was reached. Cross-sectional areas and fibre type proportions were measured in 500 fibres from six non-over-lapping fields at x40 view from six muscles per group.

### Transmission electron microscopy

Fresh tissue samples of intercostal muscles were fixed in 2% glutaraldehyde (TAAB Lab), osmicated in 1% osmium tetroxide (Agar Scientific), dehydrated and embedded in epoxy resin (Epoxy embedding resin kit, TAAB Lab). Semi-thin survey sections of 0.5 μm were cut and stained with 1% toluidine blue in 1% borax. Ultrathin sections (70 nm approximately) were then cut and stained with 2% aqueous Uranyl Acetate and Lead Citrate (Leica). The grids were examined on a Hitachi HT7800 transmission electron microscope using an Emsis Xarosa camera with Radius software. Four intercostal muscles from three animals of each group (salbutamol treated ColQ^−/−^, water treated ColQ^−/−^ or WT) were subjected to EM analysis. Quantitative analysis was done with 15 or more electron micrographs analysed by ImageJ. At each distinct region of postsynaptic folding the following features were measured, as described by Slater et al (43): nerve terminal area (total area of axon terminal); presynaptic length (total length of the nerve terminal in direct contact with the muscle fibre); postsynaptic area (total area containing postsynaptic folds); postsynaptic length (total surface length of subneural apparatus measured along the tops of the folds); fold length (total length of postsynaptic membrane including folds); fold number (the number of distinct postsynaptic folds). These measurements were used to calculate the following derived variables: (i) Occupancy (presynaptic length/postsynaptic length); (ii) fold index (fold length/postsynaptic length); (iii) fold density (fold length/postsynaptic area).

### Statistical analysis

Data are expressed as means ± S.D. unless otherwise stated. Statistical analyses were performed using GraphPad Prism Version 7 (GraphPad, San Diego, CA, USA) by pair-wise comparisons between 2 conditions with unpaired Student’s *t* tests or Mann-Whitney *U* tests. We confirmed normal distributions of data before performing parametric tests using the D’Agostino Pearson omnibus normality test. *P* < 0.05 denoted significance. Datasets were tested for outliers using the ROUT method (robust regression and outlier removal; Q  =  1%). None of the outliers affected statistical significance and all were included in analysis. Images were analysed in a blinded fashion by the same experimenter.

### Data availability

The authors confirm that the data supporting the findings of this study are available within the article and its Supplementary material. Inquiries for additional data are available from the corresponding author, upon reasonable request.

## Supplementary Material

Supplemental_Fig_1_ddz059

Supplemental_Table 1_ddz059
