# Supplementary figures and images for: Salbutamol modifies the neuromuscular junction in a mouse model of ColQ myasthenic syndrome
Source: Hum Mol Genet. 2019 Apr 1;28(14):2339–51. doi: 10.1093/hmg/ddz059 (PMC6606850; doi:10.1093/hmg/ddz059)

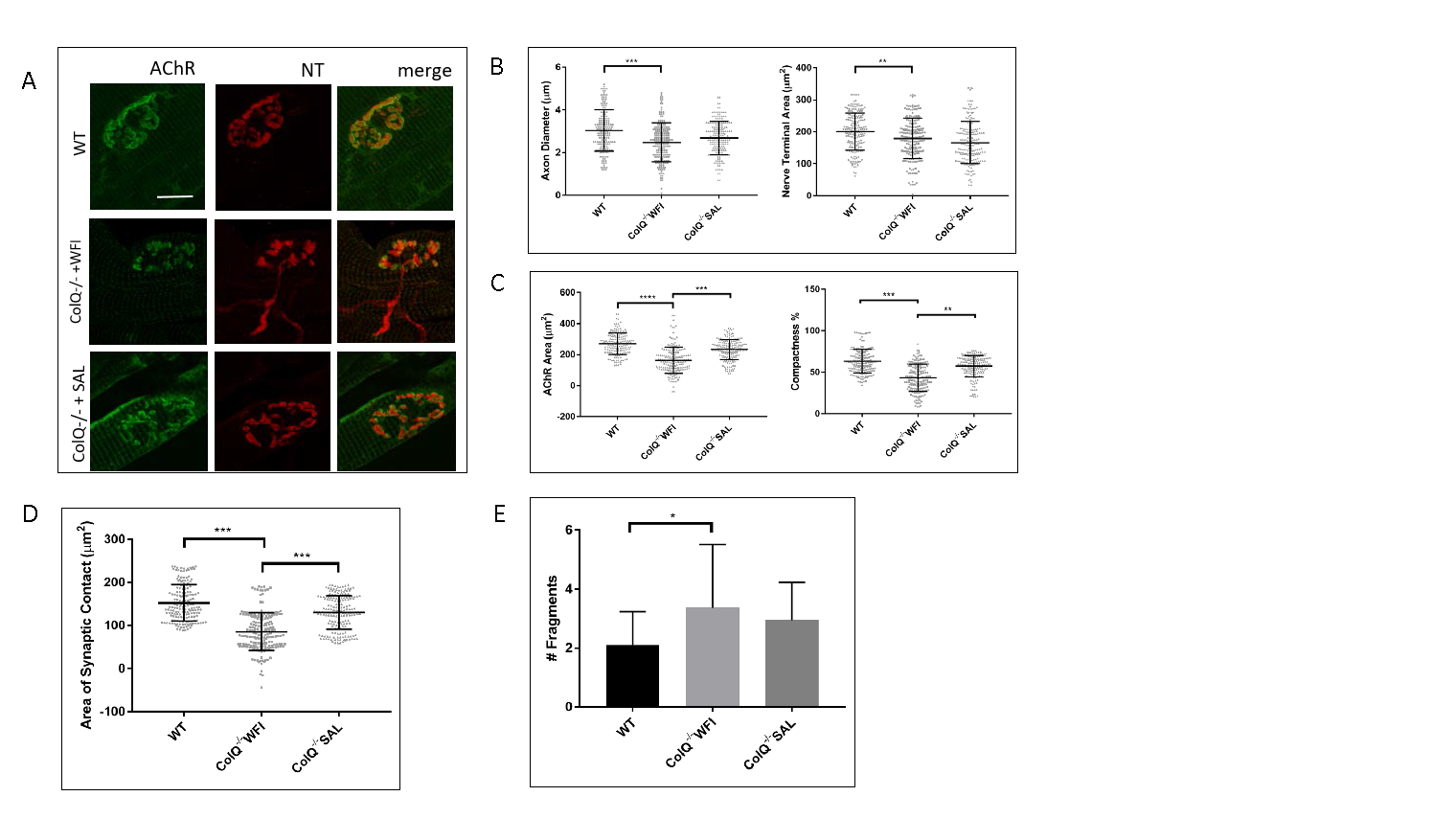

Supplement: Supplemental_Fig_1_ddz059 [file supplemental_fig_1_ddz059.png]
