# Supplementary material for: Salbutamol modifies the neuromuscular junction in a mouse model of ColQ myasthenic syndrome
Source: Hum Mol Genet. 2019 Apr 1;28(14):2339–51. doi: 10.1093/hmg/ddz059 (PMC6606850; doi:10.1093/hmg/ddz059)
Supplement: Supplemental_Table 1_ddz059 [file supplemental_table_1_ddz059.docx]

|  | Class | NTA | PreL | % Occ | PostA | PostL | FoldL | Fold Index | Fold Conc |
| --- | --- | --- | --- | --- | --- | --- | --- | --- | --- |
| Ave | WT | 7.60 | 4.53 | 0.64 | 6.49 | 7.02 | 27.93 | 4.28 | 5.11 |
| SD |  | 6.28 | 2.93 | 0.20 | 7.02 | 5.16 | 15.87 | 1.38 | 1.64 |
| N |  | 22 | 22 | 22 | 22 | 22 | 22 | 22 | 22 |
| Ave | ColQ-/- WFI | 4.53 | 3.22 | 0.73 | 3.17 | 4.45 | 11.06 | 2.50 | 3.94 |
| SD |  | 2.19 | 1.15 | 0.14 | 1.70 | 1.51 | 4.38 | 0.72 | 1.68 |
| N |  | 15 | 15 | 15 | 15 | 15 | 15 | 15 | 15 |
| *P* |  | n.s. | n.s. | n.s. | <0.05 | <0.05 | <0.001 | <0.001 | n.s. |
| Ave | ColQ-/- SAL | 3.94 | 3.11 | 0.62 | 4.16 | 5.00 | 17.98 | 3.65 | 4.67 |
| SD |  | 2.03 | 0.76 | 0.10 | 1.65 | 0.93 | 4.79 | 1.03 | 1.59 |
| N |  | 16 | 16 | 16 | 16 | 16 | 16 | 16 | 16 |
| *P* |  | n.s. | n.s. | n.s. | n.s. | n.s. | <0.01 | <0.01 | n.s. |
